# Mechanisms and Potential Treatment Options of Heart Failure in Patients With Multiple Myeloma

**DOI:** 10.7759/cureus.15943

**Published:** 2021-06-26

**Authors:** Ekaterina Proskuriakova, Keji Jada, Sandrine Kakieu Djossi, Anwar Khedr, Bandana Neupane, Jihan A Mostafa

**Affiliations:** 1 Internal Medicine, California Institute of Behavioral Neurosciences & Psychology, Fairfield, USA; 2 Research, California Institute of Behavioral Neurosciences & Psychology, Fairfield, USA; 3 Psychiatry, Psychotherapy and Research Field, California Institute of Behavioral Neurosciences & Psychology, Fairfield, USA

**Keywords:** multiple myeloma, heart failure, proteasome inhibitors, doxorubicin, immunoglobulin light-chain amyloidosis, carfilzomib, bortezomib

## Abstract

Multiple myeloma is a pathology of plasma cells, with one of the most common side effects of its treatment is heart failure. In addition, cardiac amyloidosis could cause heart failure by itself. Even though mechanisms of cardiac amyloidosis are known, and they involve lysosomal dysfunction, reactive oxygen species (ROS) accumulation, and infiltrative effect by fibrils, there is no specific agent that could protect from these effects. While the molecular mechanism of doxorubicin cardiotoxicity via topoisomerase II β is established, the only FDA-approved agent for treatment is dexrazoxane. Liposomal doxorubicin can potentially improve response and decrease the development of heart failure due to microscopic liposomes that can accumulate and penetrate only tumor vasculature. Supplements that enhance mitochondrial biogenesis are also shown to improve doxorubicin-induced cardiotoxicity. Other agents, such as JR-311, ICRF-193, and ursolic acid, could potentially become new treatment options. Proteasome inhibitors, novel agents, have significantly improved survival rates among multiple myeloma patients. They act on a proteasome system that is highly active in cardiomyocytes and activates various molecular cascades in malignant cells, as well as in the heart, through nuclear factor kappa B (NF-kB), endoplasmic reticulum (ER), calcineurin-nuclear factor of activated T-cells (NFAT), and adenosine monophosphate-activated protein kinase (AMPKa)/autophagy pathways. Metformin, apremilast, and rutin have shown positive results in animal studies and may become a promising therapy as cardioprotective agents. This article aims to highlight the main molecular mechanisms of heart failure among patients with multiple myeloma and potential treatment options to facilitate the development and research of new preventive strategies. Hence, this will have a positive impact on life expectancy in patients with multiple myeloma.

## Introduction and background

Multiple myeloma is a disorder of plasma cells that represents around 10% of all hematologic neoplasms. Its clinical features include hypercalcemia, renal problems, anemia, and bone lytic lesions [1]. The median age of patients with multiple myeloma is 65 years, and they frequently have cardiac problems when diagnosed with cancer [2].

In a retrospective study where 32,000 people with multiple myeloma were involved, nearly two-thirds of people had cardiac events at a baseline, and around 70% of patients developed cardiovascular problems during the next six years. Moreover, one of the most common adverse effects was congestive heart failure [3]. However, there were no data about the exact ejection fraction of those patients in this study.

Patients may have age-related heart problems, including thickening the heart's wall and decreasing the left ventricle size by concentric or eccentric remodeling processes [2]. The development of cardiac amyloidosis and the use of anthracyclines or proteasome inhibitors could deteriorate these processes [4].

Multiple myeloma is associated with light-chain amyloidosis (AL), which causes both toxic and infiltrative effects on the heart [5]. The infiltrative effect is associated with amyloid cardiomyopathy, characterized by the accumulation of fibrils in atria, ventricles, and vessels of the heart and causing impaired cell metabolic processes. Toxic mechanisms of cardiac amyloidosis lead to heart failure through impaired autophagy, accumulation of reactive oxygen species (ROS), and disturbance of mitochondria and lysosomes [4]. If patients with amyloid heart involvement are not treated, the survival period is approximately six months [6].

As cardiomyocytes are less prone to the regeneration processes, they are more susceptible to developing the irreversible and dose-dependent effects of agents such as doxorubicin (DOX) [7]. The mechanism of this drug was traditionally thought to be due to Fe-mediated production of ROS, which harmed cardiomyocytes as they do not have enough protection via antioxidant mechanisms. Nevertheless, the recent data reported that the drug interaction with topoisomerase II β in cardiomyocytes is much more critical [7].

Other newer drugs in treating multiple myeloma, proteasome inhibitors, such as bortezomib and carfilzomib, can also induce cardiotoxicity [8]. Carfilzomib is an irreversible proteasome inhibitor with a profound grade three to four cardiotoxic effect in 5% to 10% of patients [9]. Proteasome inhibitors work through the ubiquitin-proteasome pathway (UPP), the mechanism that controls cell survival and apoptosis [10]. Cardiomyocytes do not proliferate, and they have a much more active ubiquitin/proteasome system compared to other tissues. Therefore, by inhibiting proteasomes in the cardiomyocytes, this class of drugs causes proteasome impairment and cell apoptosis, leading to the development of heart failure [10]. According to the ENDEAVOR study (phase three study with carfilzomib and dexamethasone versus bortezomib and dexamethasone for relapsed multiple myeloma patients), the usage of carfilzomib was associated with higher rates of heart failure compared to the bortezomib group (4.8% vs. 1.8%) [11]. Although bortezomib's toxic effect on the heart has been reported to be less, it still is associated with congestive heart failure and reversible cardiac dysfunction [12].

The diagnosis of heart failure will be associated with symptoms such as fatigue, dyspnea or syncope, physical exam (regurgitations of the valves), or laboratory findings (BNP > 400 pg/mL) [13]. Cardiac imaging studies are usually performed before starting treatment in patients with multiple myeloma to assess their cardiac function [13].

This article will discuss the main mechanisms and potential treatment options of heart failure in patients with multiple myeloma, especially the effect of proteasome inhibitors (carfilzomib and bortezomib), anthracyclines (DOX), and light-chain amyloidosis. Studying these mechanisms of development of heart failure in patients with multiple myeloma is crucial as it will help to research and find new solutions to protect patients from cardiotoxic effects. Thus, it will prolong life expectancy in patients with multiple myeloma.

## Review

Mechanisms of heart failure in patients treated with proteasome inhibitors: bortezomib and carfilzomib

Proteasome inhibitors significantly improved survival rates in multiple myeloma patients. Bortezomib was the first drug approved by the US Food and Drug Administration (FDA) in 2003. After that, two other drugs were approved, carfilzomib in 2012 and ixazomib in 2015, and bortezomib became the first-line treatment for multiple myeloma [14].

In a meta-analysis assessing carfilzomib, where 2,607 people with relapsed and refractory multiple myeloma (RRMM) were included, it was shown that all grade congestive heart failure developed in 5.5% of patients. This study concluded that carfilzomib in RRMM patients increased the risk of heart failure but not ischemic heart disease compared with control [15]. In phase three FOCUS trial where patients were randomly assigned to carfilzomib or low-dose corticosteroids with optional cyclophosphamide, it was shown that carfilzomib was associated with a higher prevalence of cardiac events, including cardiac failure (7% vs. 2%) [16]. Phase three ASPIRE trial, where patients were randomly assigned to carfilzomib/lenalidomide/ dexamethasone or lenalidomide/dexamethasone, confirmed similar results and showed the development of heart failure in 3.8% in carfilzomib group compared to 1.8% in control [17].

In another meta-analysis of 25 prospective phases II and III trials, which assessed bortezomib in the treatment of various cancers, it was shown that it does not increase the potential risk of cardiovascular side effects [18]. Therefore, the incidence of heart failure in patients treated with bortezomib is predominately based on single case reports presenting this issue, whereas it is not a common finding in large studies.

Proteasome inhibitors work through binding to the β5 (chymotrypsin-like) as well as β5i proteasome subunits of the UPP, the mechanism by which the cell regulates the level of proteins and controls cell survival and apoptosis [10]. Cardiomyocytes do not proliferate, and they have a much more active ubiquitin/proteasome (UPS) system compared to other tissues. Therefore, this system plays an essential part in the heart, and proteasome impairment is associated with cardiomyopathies and heart failure [19].

Herrman et al. showed that when injecting proteasome inhibitor MLN-273 in the heart of a three-month-old healthy pig, it had a 77% lower level of chymotrypsin-like proteasome activity and a higher level of ubiquitinated proteins in contrast to the control group. In addition, their heart had evidence of hypertrophic-restrictive cardiomyopathy [20]. Hence, it is understandable that this group of drugs can cause heart failure; however, its mechanisms are still unclear.

There are several mechanisms of how proteasome inhibitors induce the death of myeloma cells. As they can affect both constitutive and immunoproteasome, this can be a reason why they also act on heart cells [21]. Proteasome inhibitors could activate heart failure in the heart also by activation of various signaling cascades.

One of the known mechanisms of how proteasome inhibitors affect myeloma cells is by blocking the pro-survival transcription factor nuclear factor kappa B (NF-kB) pathway, which regulates genes of angiogenesis, inflammation, and cell growth. As proteasomes do not degrade IκBα, it remains attached to NF-kB and prevents activation of this pathway [22]. In the heart, the role of the NF-kB has been researched for over 20 years.

Gordon et al. found that though this pathway can be protective in a short period of time by preventing apoptosis, activation over a more extended period of time can lead to heart failure via the promotion of chronic inflammation [23]. This means that in the heart, proteasome inhibitors, whose one of the mechanisms is via NF-kB, could lead to heart failure.

Rivera-Serrano et al. later showed that the NF-kB pathway is specific to a cell type in the heart and can result in a protective or damaging cardiac response. For example, cardiomyocytes are recalcitrant to the activation of this pathway. However, cardiac fibroblasts are stimulated by NF-kB, expressing cytokines of inflammation [24].

Another mechanism, promoting myeloma cell death, is associated with an endoplasmic reticulum (ER), which controls the quality of proteins that are folded incorrectly and targets those that proteasomes will disrupt. Proteasome inhibitors block the transfer of these misfolded proteins from ER to the proteasome and increase stress on ER, causing unfolded protein response (UPR), arresting cell cycle, and causing apoptosis [25].

A study by Fu et al. discussed the role of proteasome inhibitors in the ER-initiated death of cardiomyocytes. Using rat neonatal cardiomyocytes, they confirmed that proteasome inhibitors (MG132 and epoxomicin) caused ER stress-induced death of cardiomyocytes rats H9c2cells. They confirmed the finding showing activation of the ER-induced transcriptional factor ATF6 and ER-initiated apoptosis pathway via including cytosine-cytosine-adenine-adenine-thymine enhancer-binding protein (C/EBP) homologous protein (CHOP), c-Jun-N-terminal kinase (JNK), and caspase-12, which were activated by the use of proteasome inhibitors [26].

In another study conducted by Nowis et al., it was shown that bortezomib block complex V of mitochondrial respiratory chain, resulting in a significant decline in ATP production in the rat cardiomyocytes. It is an important finding as it means that heart failures caused by bortezomib through impairment of mitochondria are reversible as irreversible changes are consistent with the lack of apoptosis during histopathologic examinations. However, the heart cells' primary function is contracting that needs high levels of ATP. Therefore, cardiomyocytes are extremely sensitive to disruption of mitochondria function [27].

Tang et al. have found transactivation of the calcineurin and nuclear factor of activated T-cells (NFAT) pathway in mouth hearts and cultured adult mouse cardiomyocytes by inhibiting 20S proteasome by a pharmacological agent [28]. These findings mean that the calcineurin-NFAT pathway in the heart could be degraded and activated by proteasome inhibitors, leading to remodeling of the cells and causing heart failure.

A study conducted by Hasinoff et al. showed that carfilzomib cardiotoxicity is caused by apoptosis and cell death induction. In addition, it was found that non-toxic concentrations of proteasome inhibitors increased the disturbance of cardiomyocyte function by DOX [29]. It is an important finding as it prevents using these agents together as they may cause additive adverse effects on the heart.

In recent studies by Efentakis et al. on mice, it was found that carfilzomib may decrease the function of myocytes by increasing protein phosphatase 2 (PP2A) and by inhibiting the adenosine monophosphate-activated protein kinase (AMPKa)/autophagy pathway [30]. This mechanism was not present in bortezomib treatment, meaning that various proteasome inhibitors could have their own pathways of developing heart failure. Table 1 shows the published studies about the mechanisms of heart failure caused by proteasome inhibitors.

**Table 1:** Known studies and their results about molecular mechanisms of proteasome inhibitors-induced heart failure ER - endoplasmic reticulum, CHOP - cytosine-cytosine-adenine-adenine-thymine enhancer-binding protein (C/EBP) homologous protein, NFAT - nuclear factor of activated T-cells, NF-kB - nuclear factor kappa B, AMPKa - AMP-activated protein kinase, PP2A - protein phosphatase 2

Author	Year	Pathway	Result
Fu et al. [22]	2008	ER-stress via CHOP dependent pathway	Confirmed that proteasome inhibitors caused ER stress-induced death of rat's cardiomyocytes
Nowis et al. [23]	2010	Complex V of mitochondria respiratory chain	Bortezomib blocks complex V of mitochondria respiratory chain, resulting in a significant decline in ATP production in rat's cardiomyocytes
Tang et al. [24]	2010	Calcineurin-NFAT pathway	The calcineurin-NFAT pathway in the heart could be degraded and activated by proteasome inhibitors, leading to remodeling of the cells and causing heart failure
Gordon et al. [19]	2011	NF-kB pathway	NF-kB activation over an extended period of time promotes heart failure via signals that trigger chronic inflammation. Time and a cellular context explain the different outcome ﻿of NF-κB pathway in the heart
Rivera-Serrano et al. [20]	2017	NF-kB pathway	The heart's NF-kB pathway is specific to a cell type and can result in a protective or damaging cardiac response
Efentakis et al. [26]	2019	AMPKa/autophagy pathway	Carfilzomib may decrease the function of myocytes by increasing PP2A and by inhibiting AMPKa/autophagy pathway.

Potential strategies for heart failure prevention in patients treated with proteasome inhibitors

There are various pathways that promote the development of heart failure in patients treated for myeloma. Understanding and researching these mechanisms of cardiovascular complications could lead to the development of new ways of treatment and prevention of these side effects.

It was found that restoring AMPKa, regulating autophagy, and inhibiting apoptosis could become a promising therapy as a cardioprotective agent. In this study, the protective effect of metformin did not interact with proteasome inhibition. By mTOR complex 1, it managed to restore autophagy in cardiomyocytes. In the heart, cellular contraction could be maintained mainly by autophagy as the protein aggregates could not be degraded by proteasome mechanism [30].

Among other pharmacological treatments, it was proven by Faisal Imam et al. that apremilast has protective functions against carfilzomib toxicity. Apremilast is a phosphodiesterase-4 inhibitor that was approved for the management of psoriasis as it has potent anti-inflammatory activity [31]. In this study, the concentration of heart malondialdehyde (MDA), cardiac glutathione (GSH), and catalase (CAT) activity were measured as markers of oxidative stress. It was shown that the decreased level of GSH and CAT activity and increased concentration of MDA was reversed by treatment of apremilast. Therefore, apremilast decreases oxidative stress in cardiomyocytes. In addition, NF-kB and MAPK cascades play a significant role in carfilzomib-induced heart failure. It was shown that apremilast also reversed levels of initially increased NF-kB and extracellular signal-regulated kinase (ERK) and c-Jun NH2-terminal kinase (JNK), which are central MAPK cascades [32].

In addition, studies on animals have confirmed that rutin, or vitamin P, was able to reverse cardiotoxicity caused by carfilzomib. It was shown that rutin prevented activation of the NF-kB by increased expression of IκB-α. In addition, rutin also affects the oxidative system by changing levels of MDA and GSH. It was confirmed that it works through the reduction of hypertrophic changes as well as oxidative stress in rats [33].

There are available treatment options that have already succeeded in inhibiting pathways responsible for the development of heart failure in animal models. Therefore, these agents could become potential research objects for further investigation of drugs, which could prevent the development of heart failure in patients treated for multiple myeloma.

Molecular basics of DOX-induced heart failure and known cardioprotective agents

Despite developing and approving new drugs for treating multiple myeloma, the late-stage relapsed or refractory condition remains difficult. Those who progress on standard therapy may add to their treatment regime anthracyclines such as DOX even though its dose-dependent cardiotoxicity could limit its use. Lefrak et al. were one of the first who researched that DOX was associated with the development of irreversible and dose-dependent heart failure [34].

It was previously thought that DOX causes toxicity via the generation of ROS. As the myocardium has many mitochondria, their impairment was associated with the accumulation of ROS formed by DOX [35]. Nevertheless, another hypothesis was confirmed by Zhang et al. In their study, they showed that by deleting the Top2β gene, which encoded topoisomerase II b, they protected cardiomyocytes from DNA breaks caused by DOX and protected from ROS formation. Hence, by deletion of this gene, they protected the heart from developing heart failure [36]. Topoisomerase is an enzyme present in all dividing cells and plays a role in transcription and replication processes. There are two forms of topoisomerase2: Top2α and Top2β. It was shown that the therapeutic effect of DOX in cancer cells is associated via inhibition of Top2α, whereas the cardiotoxic effect is mediated through Top2β [37].

Inhibiting Top2 leads to a break of a double-stranded DNA that causes cell death via apoptosis and activation of the tumor suppressor gene p53 [38]. If Top2 is inhibited for a long period of time, p53 causes inhibition of the signal transducer and activator of transcription 3 (STAT 3) [39].

**Figure 1:**
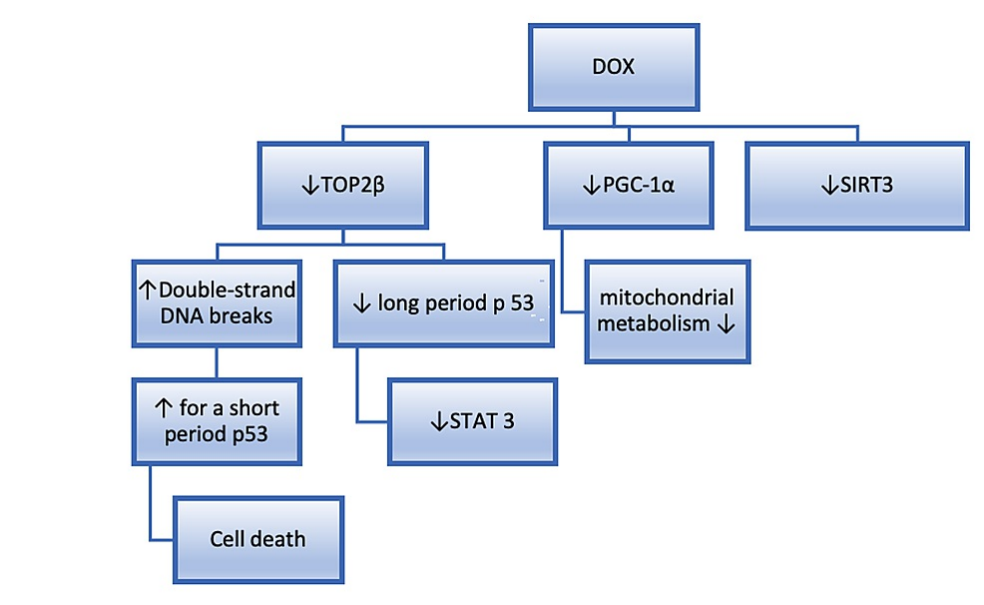
The molecular mechanisms of DOX-induced cardiotoxicity DOX - Doxorubicin, Top2β - topoisomerase2, PGC-1α - proliferator-activated receptor gamma coactivator 1-alpha, SIRT3 - Sirtuin 3, STAT 3 - signal transducer and activator of transcription 3

Another mechanism is associated with proliferator-activated receptor gamma coactivator 1-alpha (PGC-1α), which is a transcriptional coactivator of the mitochondrial metabolism [40]. Guo et al. had demonstrated on a mouse model that when treated with DOX with antioxidant metallothionein, cardiotoxicity did not develop. They have shown that the metallothionein protective effect is associated with the preservation of the activity of PGC-1α and MnSOD (manganese superoxide dismutase) [41]. Known mechanisms of DOX-induced cardiotoxicity in multiple myeloma patients are summarized in Figure 1.

Dexrazoxane (DEX), a Top2β inhibitor, is considered the only approved pharmacological substance that protects from DOX-induced cardiotoxicity in experiments and clinical practice. It prevents binding anthracyclines to topoisomerase by altering the binding site [42]. Even though there is evidence that this agent can reduce the development of heart failure, it does not improve survival or better response in patients [43]. Thus, there is a high need to research new agents that could potentially prevent and treat heart failure in patients treated with chemotherapeutic agents. 

Apart from co-administration of the DEX, several other methods have been discovered to improve cardiotoxicity caused by DOX, including the use of liposomal encapsulated forms, the development of novel anthracycline analogs, the usage of low-dose, and prolonged infusion rates [44]. Liposomal encapsulation (lipid bases) of the DOX has been considered the most successful drug-delivery strategy as liposomes cannot escape the vascular system in heart muscle where the capillary have tight junctions, whereas, in tumors cells where cells are not tightly attached, they can leave the circulation. As a result, the liposomal delivery system results in a limiting effect of DOX in the heart [44]. Even better results were achieved with pegylated liposomal formulas of DOX. Polyethylene glycol (PEG) increases the half-life of liposomal DOX (half-life: three-four days vs. 30 h for conventional formulas), leading to lower cardiovascular side effects [44]. Pegylated liposomal DOX is FDA approved in combination with bortezomib for RRMM [45]. However, this form of DOX causes significant side effect such as hand-foot syndrome (HFS) due to high affinity for the skin and long circulation time [46]. Thus, non-pegylated liposomal DOX (NPLD) is better as it has the benefits of pegylated-liposomal DOX without its disturbing side effect. Some novel DOX formulations have been extensively studied: antibody-coated liposomes, temperature-sensitive liposomes, and sulfatide-mediated liposomes. However, among all these forms, only the liposomal DOX formulations have been explored in clinical trials [44].

A novel dexrazoxane derivative JR-311 was researched in which the main mechanism of action is by degradation of Top2. Even though it preserves the pharmacodynamics of dexrazoxane, there is a problem with developing this drug due to chemical instability [47].

Recently, there was identified that a substance called ICRF-193 had much higher potency compared to DEX in terms of Top2 inhibition and cardioprotection against in vitro [48]. However, its poor water solubility prevented its in vivo administration.

Li et al. have reported in their study that one of the mechanisms of DOX toxicity is associated with the inhibition of the cardiomyocyte-autophagic flux by changing functions of lysosomes. Moreover, they have found that the degree of impairment of the autophagic flux correlates with the level of ROS production and cardiac disturbances [49]. Therefore, this finding suggests that by restoring the autophagic flux and reducing the initiation of the autophagia, cardiotoxicity caused by DOX could be prevented. One of the suggested methods of upregulation of autophagia is caloric restriction. This method has proven its efficiency in terms of cardioprotection in a rodent model after DOX usage [50].

Increasing mitochondria mass and function by elevated expression of proliferator-activated receptor gamma coactivator 1-alpha (PGC-1α) could be another mechanism to prevent DOX-induced cardiomyopathy. It could be achieved by exercise training such as endurance training for a short period of time which causes cardiac PGC-1α expression, decrease mitochondria impairment in the heart and protect from heart failure caused by DOX [51].

Another agent that improved mitochondria function via activation of Sirtuin 3 (SIRT3) was Honokiol, an extract from Magnolia Officinalis's bark. Activation of the SIRT3 pathway has been shown to improve mitochondria biogenesis and provide cardioprotection. Thus, Honokoi, being an over-the-counter supplement, can potentially be used as a post-chemotherapy agent to decrease cardiotoxic effects in patients who achieved remission [52].

L-glutamine can decrease the oxidative stress caused by DOX by increasing the amount of glutathione. In addition, it is essential for NAD+ production, which is needed for redox regulation in cells [53]. Thus, L-glutathione could improve redox potential in cells of patients that were treated with DOX.

In another study, ursolic acid (UA) was assessed as a cardioprotective treatment in mice hearts treated with DOX. It was shown that UA improved left ventricular function by increasing nitrogen oxide (NO) levels, inhibiting ROS, and decreasing apoptosis caused by DOX [54]. Hence, UA could become a new treatment option for cardiotoxicity in clinical practice induced by DOX.

There is enough evidence that supports the prominent roles of topoisomerase in DOX toxicity. Inhibition of Top2, PGC-1α, and MnSOD leads to ROS generation, which is supposed to be the secondary mechanism of DOX toxicity. Agents that improve mitochondrial function via PGC-1α and sirtuins have been effective in reversing DOX cardiotoxicity.

Light-chain amyloidosis cardiotoxicity effect in multiple myeloma

Light-chain amyloidosis (AL amyloidosis) is a disease where abnormal plasma cells secrete many light chains, which deposit in different body parts, leading to devastating injuries of the organs [55]. AL amyloidosis is considered a poor prognostic factor both for myeloma with symptoms and smoldering myeloma because of the involvement of the organs, especially the heart [56].

In a recent study conducted by Yu et al., it was confirmed that among multiple myeloma patients with AL amyloidosis, involvement of the heart had an adverse effect on survival (six months vs. 40 months). This study supported the idea that heart failure caused by AL amyloidosis is the main factor for decreasing survival among this group of patients [57].

AL amyloidosis can have a toxic and infiltrative effect on cardiomyocytes. It was shown that light-chain deposition leads to an increased level of ROS and activation of hem oxygenase in rat hearts with contractile impairment. Antioxidants stopped this pathologic mechanism [58]. Light chains cause lysosomal dysfunction leading to ROS accumulation, cellular calcium homeostasis disturbance, and cell death [59]. However, current data suggest that amyloid fibrils cause impairment of cell metabolic processes by binding themselves to the cardiomyocytes [60].

McWilliams-Koeppen et al. have demonstrated that fibrils attached to cell surface cause a decline in NAD(P)H-dependent oxidoreductase activity, an increase in oxygen consumption, and formation of the oxygen species [60]. This study confirmed that mortality and morbidity associated with cardiac amyloidosis could be without apoptosis or cell death.

Patients with AL amyloidosis and multiple myeloma have known mechanisms of the development of heart failure. However, there are not enough data about preventable treatment options from this condition compared to cardiotoxicity that proteasome inhibitors and DOX cause.

Diagnosis, monitoring, and treatment of heart failure in patients with multiple myeloma

Cardiac imaging studies are usually performed before starting treatment in patients with multiple myeloma to assess their cardiac function. There are no specific characteristics of multiple myeloma in imaging studies. However, there are some features of cardiac amyloidosis that could be helpful in the diagnosis and management. Low voltage ECG can precede heart failure and an increase in thickness of the left ventricular wall seen in an echocardiogram [13]. Echocardiography is the non-invasive test of choice, and its findings include diastolic dysfunction with high ejection fraction and increased thickness of the left ventricle wall [61]. While conventional echocardiography can capture the thickening motion of myocardial fibers that are radially oriented, it cannot assess longitudinal and circumferential fibers of the heart. Thus, it cannot differentiate between active and passive changes of the myocardium. New imaging methods, speckle-tracking echocardiography (STE), and tissue-Doppler imaging (TDI) allow physicians to notice the difference in the heart function, measured by strain and strain rate before they can see changes in the left ventricular ejection fraction [62]. STE has proven to be effective and useful in the diagnosis and prognosis of infiltrative cardiomyopathies such as cardiac amyloidosis [62]. Magnetic resonance imaging (MRI) detects global transmural to subendothelial late gadolinium enhancement. A cardiac biopsy is a gold standard for diagnosis [61].

Monitoring cardiac function using echocardiography and ECG during treatment of multiple myeloma is also recommended. A decrease in left ventricle ejection fraction (LVEF)  ≥ 10% to a value below the lower limit of normal ( ≤ 55%) should lead to the introduction of the treatment for left ventricle impairment with angiotensin-converting enzyme inhibitors (ACEI) and β-blockers [63].

Cardiac biomarkers showed to be valuable in the identification and monitoring of cardiotoxicity caused by chemotherapy. Cadinale et al. researched the role of cardiac troponin I in patients who were taking high-dose chemotherapy. They have found that an increase in cardiac troponin I is associated with the development of ventricular systolic impairment in those who were receiving chemotherapy [64]. Cardiac troponins and pro-B-type natriuretic peptide (NT-proBNP) are concurrent with increased left ventricular diastolic pressure in patients with amyloidosis [65]. While troponins and NT-proBNP are the most common biomarkers for monitoring and assessing DOX-induced cardiotoxicity, there are other markers that have been studied to predict cardiac problems in those who receive chemotherapy, such as high-sensitivity CRP (hs-CRP), which is a marker of inflammation, myeloperoxidase (MPO), a marker of oxidative stress, placental growth factor (PDF), a mediator of angiogenesis and IL-6 [66].

Treatment of heart failure in patients with multiple myeloma is challenging because of the high age of the patients, concerns for interactions between drugs, and changes in the cardiovascular system associated with age, such as orthostatic hypotension [67].

Prevention and treatment of heart failure induced by proteasome inhibitors are mainly based on the management of modifiable risk factors, decreasing the administration dose, or temporary cessation of the drug when there are severe cardiovascular side effects [68]. In addition, the usage of ACEI, angiotensin II receptor blockers (ARBs), as well as beta-blockers was shown to be effective in controlling the development of interstitial fibrosis, decreasing intracellular oxidative stress, and improving intracellular calcium metabolism, which could potentially prevent the development dysfunction of the left ventricular and heart failure [68]. Based on these findings, these medications are recommended to be administered to patients with proteasome inhibitors cardiotoxicity. In addition, it was confirmed that drugs, such as carvedilol, ARBs, and DEX, are beneficial in patients with heart failure induced by anthracyclines [69]. Recently, Bosch et al. showed that enalapril and carvedilol in patients with malignant hematologic conditions could prevent left ventricular systolic impairment [70].

General guidelines on the treatment of heart failure are published by the European Society of Cardiology (ECS) [71]. ACEI and beta-blockers are recommended as a first-line treatment for patients with heart failure symptoms with reduced ejection fraction. If those patients continue to have symptoms with LVEF ⩽ 35%, a mineralocorticoid receptor antagonist should be added. The second option for the same patients will be neprilysin or ivabradine. An implantable cardioverter-defibrillator and cardiac resynchronization may be suggested for those who remain to have LVEF ⩽ 35%, despite receiving optimal medication. Diuretics may be used to improve symptoms of fluid overload [71].

Even though there are some options to treat heart failure in patients with multiple myeloma, there is no specific medication to prevent this side effect in those patients. That is why more studies about treatment and prevention of heart failure are needed, as there are thousands of people each year who are diagnosed and treated with chemotherapy and proteasome inhibitors for multiple myeloma.

Limitations

While we were searching material for this review, there were several limiting factors. Our data were primarily obtained from the free access articles only in the English language; therefore, some articles of closed access and written in other languages may have been missed. This article highlighted the main molecular mechanisms of the development of heart failure with potential treatment strategies and did not discuss the monitoring and treatment of heart failure in detail.

## Conclusions

Modern multiple myeloma therapy has significantly improved the survival rates of patients. Multiple myeloma in patients with heart failure can complicate the management of cardiologic pathology and vice versa. Several new and established therapy agents, such as proteasome inhibitors, bortezomib, and carfilzomib, are associated with cardiotoxicity, especially the development of heart failure. Cardiac dysfunction could also be caused by chemotherapeutic agents such as DOX, which usually is related to irreversible effects on the heart. Above all, coexisting cardiac amyloidosis is the major factor that causes decreasing survival rates among patients with multiple myeloma complicated by AL. Understanding the mechanisms of heart failure is essential as it allows to use these pathways as checkpoints to develop new drugs to reduce the detrimental cardiologic effect. Metformin, apremilast, and rutin inhibit the development of heart failure by carfilzomib in animal models. DEX or special pegylated liposomal forms of the DOX can reduce DOX-induced heart failure. In addition, Sirtuin, L-glutamine, or prolonged exercise training block cardiotoxicity of DOX.

As there has not been invented a specific treatment for preventing heart failure among patients with multiple myeloma, those patients should be carefully monitored, assessed for cardiovascular risk, and prophylactically treated. There are thousands of people diagnosed and treated for multiple myeloma each year. An accurate understanding of the key mechanisms of heart failure is essential. This will help scientists invent new agents to prevent mortality and morbidity from the cardiovascular side effects of these cancer therapies.
